# The Effect of *Staphylococcus* spp., *Streptococcus* spp. and Enterobacteriaceae on the Development of Whey Protein Levels and Oxidative Stress Markers in Cows with Diagnosed Mastitis

**DOI:** 10.3390/ani10091591

**Published:** 2020-09-07

**Authors:** Kamila Puppel, Aleksandra Kalińska, Magdalena Kot, Jan Slósarz, Małgorzata Kunowska-Slósarz, Grzegorz Grodkowski, Beata Kuczyńska, Paweł Solarczyk, Tomasz Przysucha, Marcin Gołębiewski

**Affiliations:** Institute of Animal Science, Warsaw University of Life Sciences, 02-786 Warsaw, Poland; kamila_puppel@sggw.edu.pl (K.P.); alexandra.kalinska@gmail.com (A.K.); magdalenakot96@wp.pl (M.K.); jan_slosarz@sggw.edu.pl (J.S.); malgorzata_kunowska_slosarz@sggw.pl (M.K.-S.); grzegorz_grodkowski@sggw.edu.pl (G.G.); beata_kuczynska@sggw.edu.pl (B.K.); pawel_solarczyk@sggw.edu.pl (P.S.); tomasz_przysucha@sggw.edu.pl (T.P.)

**Keywords:** milk, cows, *Staphylococcus* spp., *Streptococcus* spp., *Enterobacteriaceae*, whey protein

## Abstract

**Simple Summary:**

Mastitis is one of the most common diseases of high-yielding dairy cows, and over 90% of cases are caused by *Streptococcus* spp., *Enterobacteriaceae*, or *Staphylococcus* spp. Whey proteins are very important in relation to cows’ bacteriostatic and bactericidal properties. It is therefore important to determine the relationship between the content of individual proteins and the bacterial strain. This study aimed to determine the influence of *Staphylococcus* spp., *Streptococcus* spp., and *Enterobacteriaceae* on the level of bioactive whey proteins and oxidative stress markers. From the herd, 60 multiparous cows with diagnosed mastitis were selected. Samples were taken for analyses from each cow individually from each quarter and pooled, which gave 60 samples. This study has shown that the levels of whey proteins and oxidative stress markers changed depending on the bacterial strain inducing inflammation, and selected whey proteins can be a marker for the diagnosis of individual mastitis-inducing strains.

**Abstract:**

Mastitis is one of the most common diseases of high-yielding dairy cows, and over 90% of cases are caused by *Streptococcus* spp., *Enterobacteriaceae*, or *Staphylococcus* spp. Certain groups of proteins are very significant in terms of the cow’s antioxidant, bacteriostatic, and germicidal properties: lysozyme (Lz), lactoferrin (Lf), and β-lactoglobulin (BLG). This study aimed to determine the influence of *Staphylococcus* spp., *Streptococcus* spp., and Enterobacteriaceae on the secretion of bioactive whey proteins and oxidative stress markers. From the herd, 60 multiparous cows with diagnosed mastitis were selected. Samples were taken individually from each quarter and pooled, which gave 60 samples. *Enterobacteriaceae* did not affect the BLG synthesis, whereas lysozyme and lactoferrin responded to a high concentration of these bacterial strains. In the case of *Staphylococcus* spp. infection, the BLG level increased. These strains did not affect the levels of di-malonic aldehyde (MDA), lactoferrin, and lysozyme. In contrast, they were significantly influenced by *Streptococcus* spp. In summary, the levels of whey proteins and oxidative stress markers changed depending on the bacterial strain inducing inflammation. Lysozyme and lactoferrin may be markers of udder inflammation caused by *Enterobacteriaceae* and *Streptococcus* spp., whereas β-lactoglobulin may prove useful in diagnosing *Staphylococcus* spp. induced mastitis.

## 1. Introduction

Mastitis is one of the most common diseases of high-yielding dairy cows. Mastitis causes great economic losses for the farmers and has a negative influence on milk technological value. Ruegg [1] reported, that more than 130 microorganisms have been reported to infect the bovine mammary gland. Microorganisms that most frequently cause mastitis can be divided into two categories, as follows: contagious pathogens (*Staphylococcus aureus*, *Streptococcus agalactiae)*, and environmental pathogens (*Enterobacteriaceae, Streptococcus uberis*, *Streptococcus dysgalactiae*, and coagulase-negative staphylococci (NAS)) [2,3,4,5]. Contagious pathogens are spread from cow to cow, primarily during the milking process, while environmental pathogens are found throughout the habitat of dairy cows. Unfortunately, pathogens involved in the inflammation process present lower susceptibility to antibiotics, and over 90% of cases of this disease are caused by environmental bacteria [6]. Bacterial species from these groups include: *Staphylococcus* spp.—*Staphylococcus aureus* and non-aureus staphylococci (NAS); *Streptococcus* spp.—*Streptococcus dysgalactiae, Streptococcus agalactiae*, and *Streptococcus uberis*; *Enterobacteriaceae*—*Escherichia coli, Klebsiella* spp. [2,3,4,5]. Inflammation can be accompanied by the development of symptoms such as swelling, redness, udder pain, or clots in the milk (clinical mastitis), but it can also lack such symptoms and be detectable only with microbiological or biochemical analysis (subclinical mastitis; usually defined using somatic cell count) [7].

Milk contains whey proteins which are bioactive compounds. The whey protein fraction consists of four distinct groups: albumin (~20% of total protein), immunoglobulin (~2% of total protein), serum albumin (~1% of total protein) and other proteins (~2% of total protein) e.g. lactoferrin (Lf), lysozyme (Lz), lactoperoxidase [8]. β-lactoglobulin (BLG) is globular proteins that account ~80% of the total weight of whey proteins [9]. The most important property of whey proteins is their antibacterial activity [10]. They lyse bacteria by hydrolyzing peptidoglycans within the cell wall. In turn, iron is a metal needed by microorganisms for basic metabolic processes such as cellular respiration, oxygen transport, DNA synthesis, and gene regulation [11]. Even its low concentration in the bacterial environment is very important. The concentration of lactoferrin in cow’s milk ranges from 0.12–0.450 g/L. Due to its ability to bind iron, lactoferrin does not allow it to be used by microorganisms, thus inhibiting their development and exerting a bacteriostatic effect [12,13]. It also acts as a bactericidal agent, regardless of iron binding from the environment [14,15]. The lysozyme’s lytic character is closely correlated with the growth phase of the colony. Moreover, when this enzyme is added to the suspension of Gram-positive bacteria causes the lysis of *M. luteus* bacteria at a concentration of 1 µg/mL [16,17]. 

Oxidative stress is a consequence of the imbalance between oxidants and the biological ability to quickly detoxify reactive intermediates or repair damage caused to body cells [18,19,20]. This phenomenon is triggered by reactive oxygen species (ROS) that damage cells [21]. To protect itself from ROS, the body uses its enzymatic system and endogenous antioxidants: glutathione peroxidase, superoxide dismutase, glutathione reductase, uric acid, glutathione, bilirubin, cysteine, and melatonin [22]. Understanding the underlying causes of oxidative stress occurrence can effectively prevent this phenomenon and mitigate the consequences caused by free radicals. The most reliable sources are specific biomarkers of oxidative stress, like, e.g., superoxide dismutase, glutathione peroxidase, glutathione reductase, and di-malonic aldehyde (MDA) [11]. 

It is therefore important to determine the relationship between the content of individual proteins and the bacterial strain. This study aimed to determine the influence of *Staphylococcus* spp., *Streptococcus* spp., and Enterobacteriaceae on the synthesis of bioactive whey proteins (lysozyme, lactoferrin, β-lactoglobulin) and oxidative stress markers in cows with diagnosed mastitis.

## 2. Materials and Methods

All cows were handled in accordance with the regulations of the Polish Council on Animal Care, and the Care Committee reviewed and approved all procedures. The experiment was carried out at the experimental dairy farm of the Warsaw University of Life Sciences (WULS). The cows were kept in a free-stall dairy shed and fed a total mixed ration (TMR) diet. On average, cows obtain about 9000 kg of milk per lactation, with 3.40% protein and 4.30% fat content. 

Based on previous analyzes, from the herd (lactating 420 cows), 60 multiparous Polish Holstein-Friesian cows (mid-lactation; 125 ± 28 d) with diagnosed mastitis caused by one of three bacterial species: *Staphylococcus* spp., *Streptococcus* spp., and *Enterobacteriaceae* were selected. The cows were diagnosed on the basis of: cytological quality (SCC, somatic cell count), microbiological quality of milk (total count of bacteria), and reduction cultures to detect individual strains. Samples for analysis were taken from each cow individually from each quarter, and one sample (pooled sample) was prepared for further analysis. The total number of samples was 600. The samples were collected in the sterile bottle, kept in a cold box and immediately submitted to the WULS laboratory. No cows were given antibiotic treatment prior to milk sampling.

### 2.1. Chemical Analyses

The microbiological quality of milk was determined by BactoScan (Bentley, Warsaw, Poland). Cytological quality of milk was determined by Somocaunt 150 (Bentley, Warsaw, Poland).

The determination of di-malonic aldehyde level was established using Tecan’s NanoQuant Infinite M200 PRO (Tecan Austria GmbH, Grödig, Austria) analyzer at wavelength 532 nm according to the methodology described by Kapusta et al. [20]. Mean intra-assay coefficients of variation (CVs) for samples and standards were ≤2.4%, and mean inter-assay CVs were ≤2.7%.

Total antioxidant status (TAS) were established by RANDOX application using a NanoQuant Infinietie M200Pro analyzer (Tecan Austria GmbH, Grödig, Austria). ABTS^®^ incubation with peroxidase (metmyoglobin) leads to the formation of the ABTS ^+ +^ radical cation. This substance is blue-green and can be detected at a wavelength of 600 nm. The antioxidants present in the sample decrease the formation of the blue-green color, in proportion to their concentration. Mean intra-assay CVs for samples were ≤2.1% and mean inter-assay CVs were ≤2.5%.

Concentrations of whey proteins were determined using an Agilent 1100 Series RP-HPLC (Agilent Technologies, Waldbronn, Germany). Separations were performed at ambient temperature using solvent gradient on Jupiter column C18 300A (Phenomenex, Torrance, CA, USA). The chromatographic conditions were as follows. Solvent A was acetonitrile (Merck, Darmstadt, Germany), water (Sigma–Aldrich, St. Louis, MO, USA) and trifluoroacetic acid (Sigma–Aldrich, St. Louis, USA) in a ratio of 50:950:1 (*v*/*v*/*v*). Solvent B was acetonitrile, water and trifluoroacetic acid in a ratio of 950:50:1 (*v*/*v*/*v*). The flow rate was 1.2 mL/min and the detection wavelength was 220 nm. The injection volume of final solution was 25 μL. All samples were analysed in duplicate. The identification of peaks as lactoferrin and lysozyme was confirmed by a comparison with the standards: Lf and Lz (Sigma-Aldrich, St. Louis, MO, USA).

Milk samples were used in microbiological analysis using WASP analyzer (Bentley, Warsaw, Poland) for automatic cultures (50 µL from each quarter milk sample). Appropriate dilutions were transferred to sterile 90 mm Petri dishes. Mannitol Salt Lab Agar (Biomaxima, Lublin, Poland) was used to study *Staphylococcus* spp. Edwards Lab Agar (Biomaxima, Lublin, Poland) was used to study *Streptococcus* spp. spp. Chromogenic Uri-Color Lab Agar (Biomaxima, Lublin, Poland) was used to study *Enterobacteriaceae*. *Staphylococcus* spp., *Streptococcus* spp., and Enterobacteriaceae were incubated at 37 °C for 24 h. All samples were analysed in duplicate.

### 2.2. Statistical Analyses

The experimental data were statistically analyzed using SPSS 23 [23]. Data were statistically processed by applying GLM procedure with fixed effects of SCC, and bacteria strains (*Enterobacteriaceae, Staphylococcus* spp., *Streptococcus* spp.). Data presented in the tables are least square mean and standard error values.

Y_ijkl_ = μ + A_i_ + B_j_ + C_k_ + D_l_ + e_ijkl,_ (1)

where Y*_ijkl_* is the dependent variable; μ is the overall mean; A*_i_* is the fixed effect of SCC (i = 1–3); Bj is the fixed effect of *Enterobacteriaceae* (j = 1–3); Ck is the fixed effect of *Staphylococcus* spp. (k = 1–3); D_l_ is the fixed effect of *Streptococcus* spp. (l = 1–3); e*_ijk_* is the residual error.

For post hoc analysis, Duncan’s test was performed with α < 0.05 and 0.01.

The count of bacteria determined in the samples was presented in the paper as log CFU/mL of milk, i.e., the number of colony forming units in 1 mL of milk. In the statistical analysis, a division into three groups of cows was applied, taking into account the count of individual bacteria (*Enterobacteriaceae, Staphylococcus* spp., *Streptococcus* spp.):

1. 0 CFU/mL
2. 0.1–1 CFU/mL
3. >1.1 CFU/mL

The control group consisted cows that have resolved the inflammation/killed the mastitis pathogen; 0 CFU/mL which were the reference to the comparison in relation to 0.1–1.0 and >1.1 CFU/mL. 

In the statistical analysis, a division into three groups of cows was applied, taking into account the SCC:

1. <200,000 cell/mL
2. 200,000–400,000 cell/mL
3. >400,000 cell/mL

The control group consisted of <200,000 cell/mL samples which were the reference to the comparison in relation to 200,000–400,000 cell/mL and >400,000 cell/mL.

## 3. Results and Discussion

In high-yielding dairy cows, oxidative stress leads to adverse changes in the nutritional value of milk and dairy products [20]. It also has a negative impact on animal health, may cause reproductive problems and many metabolic disorders [11,24]. Bovine mastitis due to pathogen invasion, is a major concern of the dairy industry [25]. Additionally, Wessely-Szponder et al. [26] demonstrated that considerable amounts of NO and the myeloperoxidase enzyme are produced in cow’s body during the inflammatory process. Malondialdehyde modifies the physical structures of cell membranes and is indirectly involved in the synthesis of protein, DNA, and RNA [11,20]. It also features mutagenic and carcinogenic properties [27,28]. A significant correlation has been shown in the present study between MDA concentration and SCC (Table 1; *p* ≤ 0.01). The greater MDA level (28.749 nM/mL) was determined in milk having SCC > 400,000 cell/mL. An increase in MDA concentration indicates cows’ exposure to oxidative stress (Table 1). Milk with a greater somatic cell count has been shown to have more infiltrated polymorphonuclear cells, which promotes oxidative reactions [29]. Additionally, Suriyasathaporn et al. [30] reported that increased MDA concentration due to high SCC contributed to milk quality deterioration. Samples with a SCC < 200,000 were in fact taken from cows diagnosed with clinical mastitis. We are unsure if these cows had resolved the mastitis and eliminated a bacterial infection prior to sample collection, were never infected with a bacterial pathogen (the mastitis may have been the result of some other inflammatory stimulus such as an injury), or were simply misdiagnosed. Nevertheless, these samples had a low SCC.

A kit for the determination of the total level of antioxidants - TAS, enables the assessment of an integrated antioxidant system that covers all biological components, which exhibit antioxidant activity [20]. However, a high level of TAS in the milk of cows where the SCC <200,000 cell/mL indicates that healthy cows are predisposed to a higher antioxidant potential (Table 1). According to Kuczaj et al. [31], whey protein concentrations in milk from cows with mastitis are greater than those in healthy cows. This relationship was confirmed by the results obtained in the present study.

**Figure 1:**
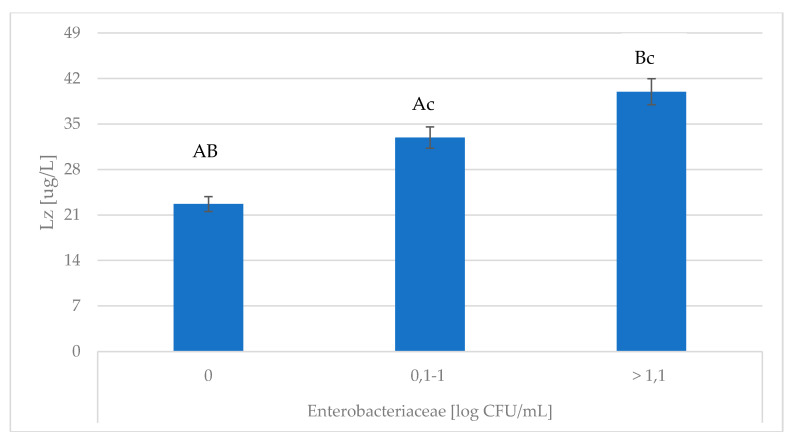
Association of *Enterobacteriaceae* with changes in Lz content. Lz—lysozyme; ^A,B,c^ Means with the same letters differ significantly at: small letters—*p* ≤ 0.05; capitals—*p* ≤ 0.01.

**Figure 2:**
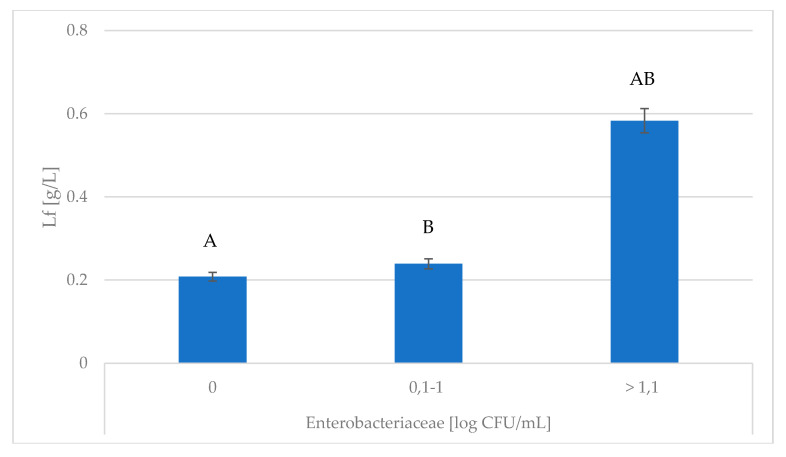
Association of *Enterobacteriaceae* with changes in Lf content. Lf—lactoferrin; ^A,B^ Means with the same letters differ significantly at: capitals—*p* ≤ 0.01.

*Enterobacteriaceae* infections with a log CFU > 1.1 were associated with the greatest degree of oxidative stress (decreasing TAS and increasing MDA content). Studies have shown that MDA concentration was significantly influenced by Enterobacteriaceae (Table 2; *p* ≤ 0.01). The greater concentrations of Lz and Lf were found in milk in which the Enterobacteriaceae count was > 1.1 log CFU/mL (Figure 1 and Figure 2). According to Zimecki and Artym [32], lactoferrin is a peptide which strongly responds to *E. coli* and *Pseudomonas aureginosa*. This relationship was confirmed by the results obtained in the present study.

**Figure 3:**
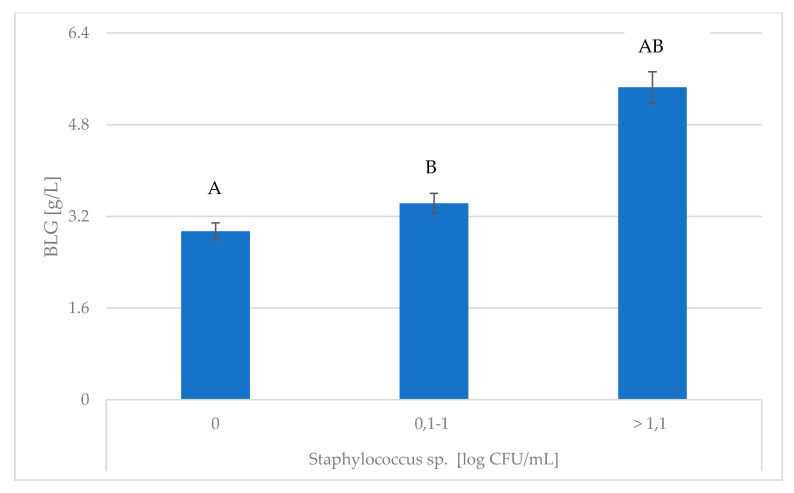
Association of *Staphylococcus* spp. with changes in BLG content. BLG—β-lactoglobulin; ^A,B^ Means with the same letters differ significantly at: capitals—*p* ≤ 0.01.

Studies have shown that the concentration of BLG was significantly influenced by *Staphylococcus* spp. (Figure 3). Waage et al. [33] reported that *Staphylococcus aureus* had been implicated in 7–44% of clinical mastitis cases. Additionally, BLG by reducing the colonization of *Staphylococcus aureus* and *Streptococcus uberis*, β-LG minimizes the risk of mastitis in the herd [7]. Mazmanian et al. [34] reported that *Staphylococcus aureus* carries receptors on its surface which are able to specifically bind a wide variety of host proteins. During *S. aureus* infection, the milk proteins showing the greatest up-regulation were antimicrobial peptides/proteins, NET proteins, and other regulators of proinflammatory innate immune responses [35].

According to Jahani et al. [36], cows infected with Gram-negative bacteria have lower lysozyme activity than Gram-positive bacteria like, e.g., *S. epidermidis* and *B. cereus*. Andrzejczak [37] claims that this is due to the presence of Gram-negative bacteria, additional polypeptides, lipoproteins, and lipopolysaccharides in cell walls. They provide additional protection in the form of a barrier that makes it difficult for enzymes to access the bacterial cell’s interior. Comparing the value of the results for Lz for a code 0 CFU/mL *Staphylococcus* spp. in Table 3 with the value it adopts in Table 2 and Table 4 for a code > 1.1 CFU/mL Enterobacteriaceae and *Streptococcus* spp., the theories of these authors can be confirmed.

**Figure 4:**
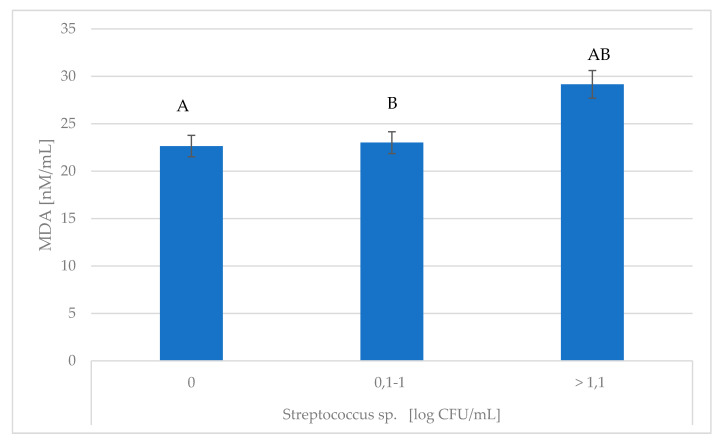
Association of *Streptococcus* spp. with changes in MDA content. MDA—di-malonic aldehyde; ^A,B^ Means with the same letters differ significantly at: capitals—*p* ≤ 0.01.

The present study showed that the concentrations of MDA, Lz, and Lf were significantly influenced by *Streptococcus* spp. (Figure 4, Table 4). 

According to Gasińska [38], lactoferrin exhibits antibacterial activity against streptococci, which is confirmed by the results of the present research. Such conclusions were also reached by Jahani et al. [36], who claimed that lactoferrin had antibacterial effect on *Streptococcus pyogenes, S. canis, S. agalactiae, Klebsiella pneumoniae, S. zooepidermicus*, and *Candidia albicans*. Studies conducted by Ellison and Giehl [39] show that lactoferrin and lysozyme kill Gram-negative bacteria synergistically. This activity can be demonstrated against a wide variety of bacteria, is dose-dependent, and depends on the size of the bacterial inoculum and basic culture media. In contact with Gram-negative bacteria, Lf combines with its surface proteins (porous), causing the release of lipopolysaccharide, which results in an increase in membrane permeability, antibacterial factors, and osmotic pressure [15]. This process is influenced by the presence of calcium, magnesium, and iron cations [40]. The breakdown of Gram-positive bacteria is due to the combination of positively-charged proteins with the bacterial membrane. Most often, it is at this stage that the bacterial cell is destroyed [15]. These effects may include the direct action of lactoferrin, as well as a change in bacterial metabolism.

## 4. Conclusions

The interaction between mastitis pathogens and the immune system is intricate, because both have the ability to co-evolve to recognize, respond, and adapt to the other. *Enterobacteriaceae* did not affect the BLG synthesis, whereas Lz and Lf responded to a high concentration of these bacterial strains. In the case of *Staphylococcus* spp. infection, the BLG level increased. These strains did not affect the levels of MDA, Lf, and Lz. In contrast, they were significantly influenced by *Streptococcus* spp. Unlike staphylococci, the level of MDA increases in the cow’s milk with a high *Streptococcus* spp. content. In summary, the levels of whey proteins and oxidative stress markers changed depending on the bacterial strain inducing inflammation. The diverse pathogens that cause mastitis induce different responses in the mammary gland, and therefore udder requires highly specific pathogen-dependent responses for protection. Lysozyme and lactoferrin may be markers of udder inflammation caused by Enterobacteriaceae and *Streptococcus* spp., whereas β-lactoglobulin may prove useful in diagnosing *Staphylococcus* spp. induced mastitis.

## Figures and Tables

**Table 1:** Association of SCC with changes in whey proteins, MDA and TAS content.

	SCC [10^3^ cell/mL]	LSM	SEM
MDA [nM/mL]	<200	19.369 ^AB^	0.151
	200–400	21.091 ^AC^	0.184
	>400	28.749 ^BC^	0.169
TAS [mmol/L]	<200	0.808 ^aB^	0.027
	200–400	0.717 ^aC^	0.028
	>400	0.471 ^BC^	0.027
Lz [µg/L]	<200	30.112 ^A^	1.270
	200–400	30.024 ^B^	1.272
	>400	34.076 ^AB^	1.278
Lf [g/L]	<200	0.239 ^A^	0.016
	200–400	0.245 ^B^	0.018
	>400	0.469 ^AB^	0.016
BLG [g/L]	<200	3.239 ^A^	0.123
	200–400	3.368 ^B^	0.103
	>400	5.333 ^AB^	0.139

^a,A,B,C^ Means in the same column marked with the same letters differ significantly at: small letters—*p* ≤ 0.05; capitals—*p* ≤ 0.01. LSM—least squares of means; SEM—standard error of mean; MDA—di-malonic aldehyde; TAS—total level of antioxidants; SCC—somatic cell count; Lz—lysozyme; Lf—lactoferrin; BLG—β-lactoglobulin.

**Table 2:** Association of *Enterobacteriaceae* with changes in MDA, TAS and BLG content.

	*Enterobacteriaceae* [log CFU/mL]	LSM	SEM
MDA [nM/mL]	0	20.630 ^AB^	1.469
	0.1–1	25.006 ^AC^	1.028
	>1.1	29.611 ^BC^	1.124
TAS [mmol/L]	0	0.806	0.041
	0.1–1	0.787	0.039
	>1.1	0.668	0.047
BLG [g/L]	0	3.383	0.176
	0.1–1	3.569	0.128
	>1.1	3.193	0.144

^A,B,C^ Means in the same column marked with the same letters differ significantly at: small letters—*p* ≤ 0.05; capitals—*p* ≤ 0.01. LSM—least squares of means; SEM—standard error of mean; MDA—di-malonic aldehyde; TAS—total level of antioxidants; BLG—β-lactoglobulin.

**Table 3:** Association of *Staphylococcus* spp. with changes in whey proteins, MDA and TAS content.

	*Staphylococcus* spp. [log CFU/mL]	LSM	SEM
MDA [nM/mL]	0	26.224 ^AB^	1.570
	0.1–1	22.034 ^A^	1.794
	>1.1	22.000 ^B^	1.418
TAS [mmol/L]	0	0.865	0.060
	0.1–1	0.868	0.060
	>1.1	0.765	0.044
Lz [µg/L]	0	34.310 ^A^	0.503
	0.1–1	29.489 ^AB^	0.240
	>1.1	31.112 ^B^	0.263
Lf [g/L]	0	0.294	0.038
	0.1–1	0.243	0.022
	>1.1	0.247	0.022

^A,B^ Means in the same column marked with the same letters differ significantly at: capitals—*p* ≤ 0.01. LSM—least squares of means; SEM—standard error of mean; MDA—di-malonic aldehyde; TAS—total level of antioxidants; Lz—lysozyme; Lf—lactoferrin.

**Table 4:** Association of *Streptococcus* spp. with changes in whey proteins and TAS content.

	*Streptococcus* spp. [log CFU/mL]	LSM	SEM
TAS [mmol/L]	0	0.899	0.057
	0.1–1	0.716	0.045
	>1.1	0.867	0.060
Lz [µg/L]	0	30.165 ^A^	1.221
	0.1–1	30.662 ^B^	1.797
	>1.1	42.118 ^AB^	1.451
Lf [g/L]	0	0.265 ^A^	0.019
	0.1–1	0.228 ^B^	0.026
	>1.1	0.474 ^AB^	0.038
BLG [g/L]	0	4.576 ^AB^	0.209
	0.1–1	3.195 ^A^	0.243
	>1.1	3.032 ^B^	0.285

^A,B^ Means in the same column marked with the same letters differ significantly at: capitals—*p* ≤ 0.01. LSM—least squares of means; SEM—standard error of mean; TAS—total level of antioxidants; Lz—lysozyme; Lf—lactoferrin; BLG—β-lactoglobulin.
